# PARK14 PLA2G6 mutants are defective in preventing rotenone-induced mitochondrial dysfunction, ROS generation and activation of mitochondrial apoptotic pathway

**DOI:** 10.18632/oncotarget.20893

**Published:** 2017-09-15

**Authors:** Ching-Chi Chiu, Tu-Hsueh Yeh, Chin-Song Lu, Yin-Cheng Huang, Yi-Chuan Cheng, Ying-Zu Huang, Yi-Hsin Weng, Yu-Chuan Liu, Szu-Chia Lai, Ying-Ling Chen, Yu-Jie Chen, Chao-Lang Chen, Hsin-Yi Chen, Yan-Wei Lin, Hung-Li Wang

**Affiliations:** ^1^ Neuroscience Research Center, Chang Gung Memorial Hospital at Linkou, Taoyuan, Taiwan; ^2^ Healthy Aging Research Center, Chang Gung University College of Medicine, Taoyuan, Taiwan; ^3^ Department of Nursing, Chang Gung University of Science and Technology, Taoyuan, Taiwan; ^4^ Department of Neurology, Taipei Medical University Hospital, Taipei, Taiwan; ^5^ School of Medicine, Taipei Medical University, Taipei, Taiwan; ^6^ Division of Movement Disorders, Department of Neurology, Chang Gung Memorial Hospital at Linkou, Taoyuan, Taiwan; ^7^ College of Medicine, Chang Gung University, Taoyuan, Taiwan; ^8^ Department of Neurosurgery, Chang Gung Memorial Hospital at Linkou, Taoyuan, Taiwan; ^9^ Graduate Institute of Biomedical Sciences, Chang Gung University College of Medicine, Taoyuan, Taiwan; ^10^ Institute of Cognitive Neuroscience, National Central University, Taoyuan, Taiwan; ^11^ Division of Sports Medicine, Taiwan Landseed Hospital, Taoyuan, Taiwan; ^12^ Department of Physiology and Pharmacology, Chang Gung University College of Medicine, Taoyuan, Taiwan

**Keywords:** Parkinson’s disease, PARK14, PLA2G6, rotenone, mitochondrial dysfunction

## Abstract

Mutations in the gene encoding Ca^2+^-independent phospholipase A_2_ group 6 (PLA2G6) cause the recessive familial type 14 of Parkinson’s disease (PARK14). Mitochondrial dysfunction is involved in the pathogenesis of Parkinson’s disease (PD). PLA2G6 is believed to be required for maintaining mitochondrial function. In the present study, rotenone-induced cellular model of PD was used to investigate possible molecular pathogenic mechanism of PARK14 mutant PLA2G6-induced PD. Overexpression of wild-type (WT) PLA2G6 ameliorated rotenone-induced apoptotic death of SH-SY5Y dopaminergic cells. PARK14 mutant (D331Y), (G517C), (T572I), (R632W), (N659S) or (R741Q) PLA2G6 failed to prevent rotenone-induced activation of mitochondrial apoptotic pathway and exert a neuroprotective effect. WT PLA2G6, but not PARK14 mutant PLA2G6, prevented rotenone-induced mitophagy impairment. In contrast to WT PLA2G6, PARK14 mutant PLA2G6 was ineffective in attenuating rotenone-induced decrease in mitochondrial membrane potential and increase in the level of mitochondrial superoxide. WT PLA2G6, but not PARK14 PLA2G6 mutants, restored enzyme activity of mitochondrial complex I and cellular ATP content in rotenone-treated SH-SY5Y dopaminergic cells. In contrast to WT PLA2G6, PARK14 mutant PLA2G6 failed to prevent rotenone-induced mitochondrial lipid peroxidation and cytochrome c release. These results suggest that PARK14 PLA2G6 mutants lose their ability to maintain mitochondrial function and are defective inpreventing mitochondrial dysfunction, ROS production and activation of mitochondrial apoptotic pathway in rotenone-induced cellular model of PD.

## INTRODUCTION

Parkinson’s disease (PD), the second most common neurodegenerative disorders, is characterized by the loss of dopaminergic neurons in substantia nigra pars compacta (SNpc) [1]. The prevalence of PD is 1-2% in elderly people over 65 years [2, 3]. The etiology of PD has been thought to result from a combination of genetic, environmental and epigenetic factors [4, 5]. Although the molecular pathogenesis of PD is not completely understood, mitochondrial dysfunction, elevated oxidative stress, inflammation and environmental toxins are involved in the pathogenesis of PD [6].

Patients affected with familial type 14 of Parkinson’s disease (PARK14) exhibit autosomal recessive inheritance and early-onset dystonia-parkinsonism [7–10]. Initial studies reported that mutations in the gene encoding Ca^2+^-independent phospholipase A_2_ group 6 (PLA2G6) is the cause of infantile neuroaxonal dystrophy (INAD) and neurodegeneration with brain iron accumulation (NBIA) [11, 12]. NBIA, according to the clinical phenotype associated with the genetic causes, is classified into pantothenate kinase-associated neurodegeneration (NBIA 1), PLA2G6-associated neurodegeneration (PLAN)/ infantile neuroaxonal dystrophy (INAD) (NBIA 2), neuroferritinopathy (NBIA 3), fatty acid hydroxylase-associated neurodegeneration (FAHN), mitochondrial membrane-associated neurodegeneration (MPAN), COASY protein-associated neurodegeneration (CoPAN), β-propeller-associated neurodegeneration (BPAN), Kufor-Rakeb syndrome and Woodhouse-Sakati syndrome [13, 14]. Subsequent investigations indicated that mutations of PLA2G6 gene also cause PARK14 [8–10, 15–19]. Numerous missense mutations of PLA2G6, including (D331Y), (G517C), (T572I), (R632W), (N659S) and (R741Q) PLA2G6, were found in PARK14 patients [7, 15–17, 19–21]. The molecular pathogenic mechanism of PARK14 mutant PLA2G6-induced PD remains unknown.

Multiple lines of evidence suggest that mitochondrial dysfunction and oxidative stress play an important role in the pathogenesis of PD [22, 23]. The loss of SNpc dopaminergic neurons in PD patients is believed to result from mitochondrial dysfunction, leading to the activation of mitochondria-mediated apoptotic pathway [6, 23–25]. A decreased activity of mitochondrial complex I and an increased level of oxidative stress products were observed in the substantia nigra of PD patients [6, 25, 26]. The administration of complex I inhibitor rotenone causes the death of dopaminergic neurons by activating mitochondrial apoptotic pathway [27–30]. PLA2G6 has been shown to be expressed in the mitochondria [31]. Expression of PLA2G6 in INS-1 cells exerts an anti-apoptotic effect by preventing staurosporine-induced mitochondrial dysfunction and apoptosis [31]. PLA2G6 deficiency in the *Drosophila* causes mitochondrial dysfunction, oxidative stress and abnormality of mitochondrial membrane [32]. Therefore, it is very likely that PLA2G6 exerts anti-apoptotic and neuroprotective effects on SNpc dopaminergic cells by playing an important role in maintaining mitochondrial function. Autosomal recessive inheritance of PARK14 suggests that PARK14 mutations cause the loss of PLA2G6 function and impair the ability of PLA2G6 to protect mitochondrial function against apoptotic stimuli and exert a neuroprotective effect on dopaminergic cells. In the present study, we tested this hypothesis by expressing wild-type or PARK14 mutant PLA2G6 in rotenone-treated SH-SY5Y dopaminergic cells, a cellular model of PD. Our results showed that in contrast to wild-type PLA2G6, PARK 14 mutant (D331Y), (G517C), (T572I), (R632W), (N659S) or (R741Q) PLA2G6 failed to prevent mitochondrial dysfunction, reactive oxygen species (ROS) production and activation of mitochondrial apoptotic pathway in rotenone-induced cellular model of PD.

## RESULTS

### PARK14 PLA2G6 mutants fail to prevent rotenone-induced death of dopaminergic cells

**Figure 1:**
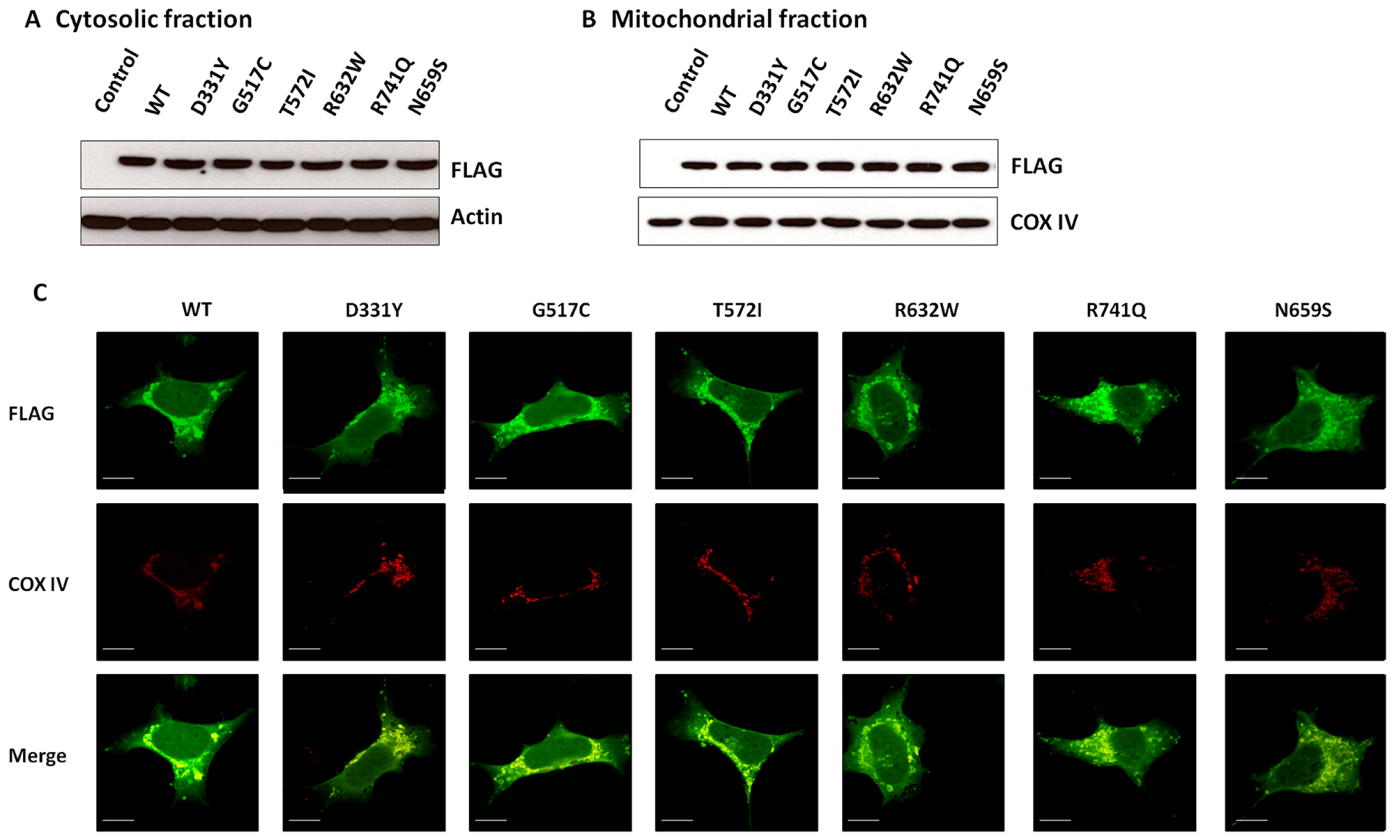
WT PLA2G6 or PARK14 mutant PLA2G6 is expressed in the cytosolic and mitochondrial fractions of SH-SY5Y dopaminergic cells **(A** and **B)** SH-SY5Y cells were transfected with cDNA of FLAG-tagged WT or PARK14 mutant PLA2G6, transfected SH-SY5Y cells were subfractionated into cytosolic (A) and mitochondrial fractions (B). Western blot analysis using anti-FLAG antibody indicated that similar to WT PLA2G6, PARK14 mutant (D331Y), (G517C), (T572I), (R632W), (N659S) or (R741Q) PLA2G6 was expressed in both cytosol and mitochondria. Cytochrome c oxidase subunit IV (COX-IV) was used as an internal control for mitochondrial fraction. **(C)** Immunofluorescence staining studies showed that FLAG-tagged WT or PARK14 PLA2G6 mutant was localized in cytosolic and mitochondrial fractions (Scale bar is 10 μm). COX-IV is a mitochondrial marker.

To visualize the subcellular distribution of wild-type (WT) or PARK14 mutant PLA2G6, SH-SY5Y dopaminergic cells were transfected with the cDNA of WT or PARK14 mutant PLA2G6. Subcellular fractionation study and immunofluorescence staining showed that similar to WT PLA2G6, PARK14 mutant (D331Y), (G517C), (T572I), (R632W), (N659S) or (R741Q) PLA2G6 was expressed in both cytosolic and mitochondrial fractions (Figure 1). A similar transfection efficiency of cDNA encoding WT or mutant PLA2G6 resulted in a similar protein expression level of WT or mutant PLA2G6 in the cytosolic or mitochondrial fraction.

**Figure 2:**
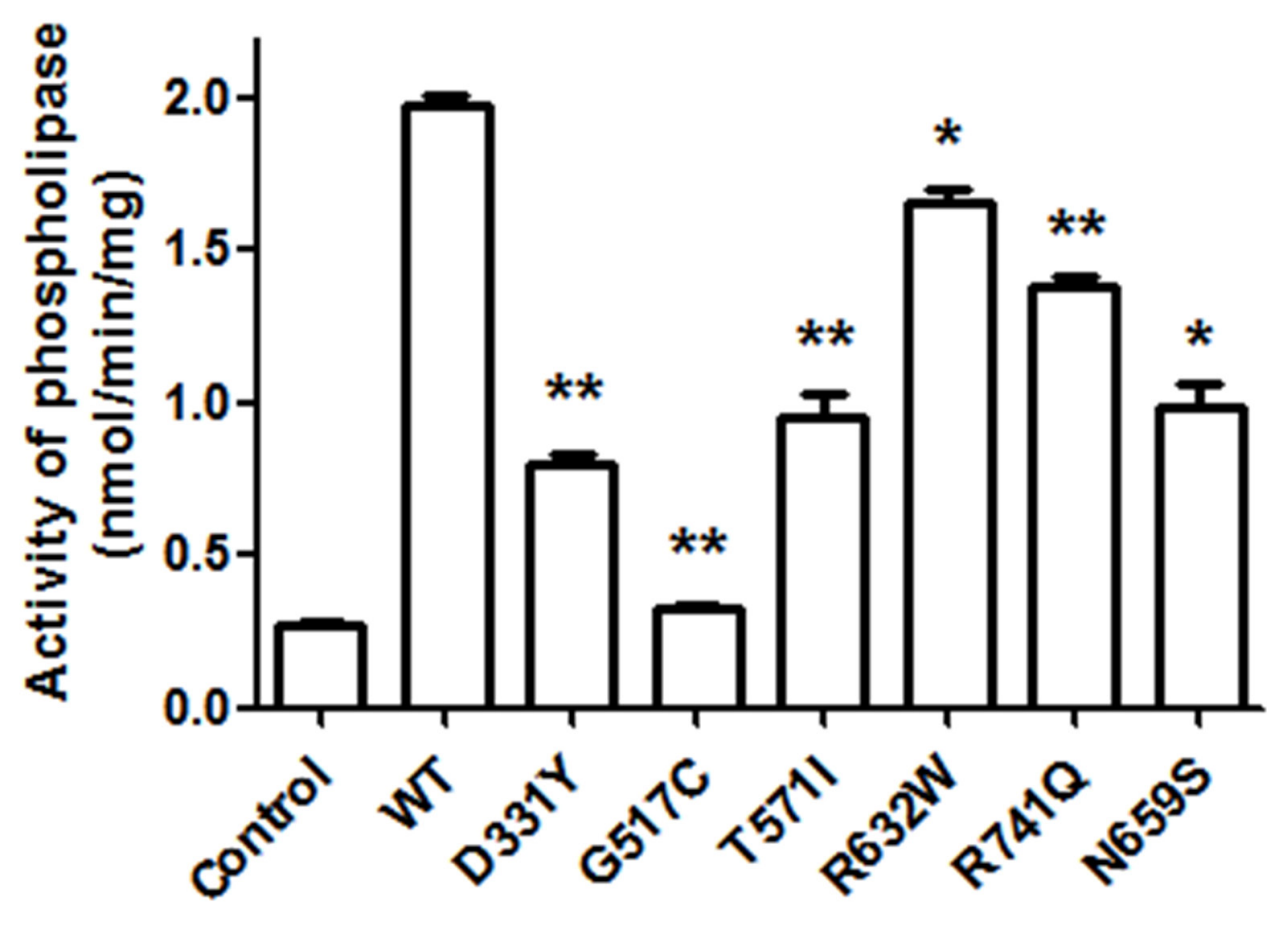
PARK14 mutant PLA2G6 exhibits an impaired enzyme activity of phospholipase A2 (PLA2) Two days after the transfection, the PLA_2_ activity of WT or PARK 14 mutant PLA2G6 expressed in SH-SY5Y cells was determined by using cPLA_2_ assay kit. Expression of WT PLA2G6 greatly increased the activity of phospholipase A_2_. Compared to WT PLA2G6, the PLA_2_ activity was significantly decreased in SH-SY5Y cells expressing PARK14 mutant PLA2G6. Each bar represents the mean ± SEM value of six independent experiments. *p< 0.05, **p<0.01 compared to SH-SY5Y cells expressing WT PLA2G6.

To study the functional consequence of PARK14 mutations on the enzyme activity of PLA2G6, we evaluated the phospholipase A_2_ activity in SH-SY5Y dopaminergic cells expressing WT PLA2G6 or PARK14 mutant PLA2G6. Compared to WT PLA2G6, PARK14 mutant (D331Y), (G517C), (T572I), (R632W), (N659S) or (R741Q) PLA2G6 exhibited a significantly reduced activity of phospholipase A_2_ (Figure 2). This finding suggests that PARK14 mutations cause the loss of function.

**Figure 3:**
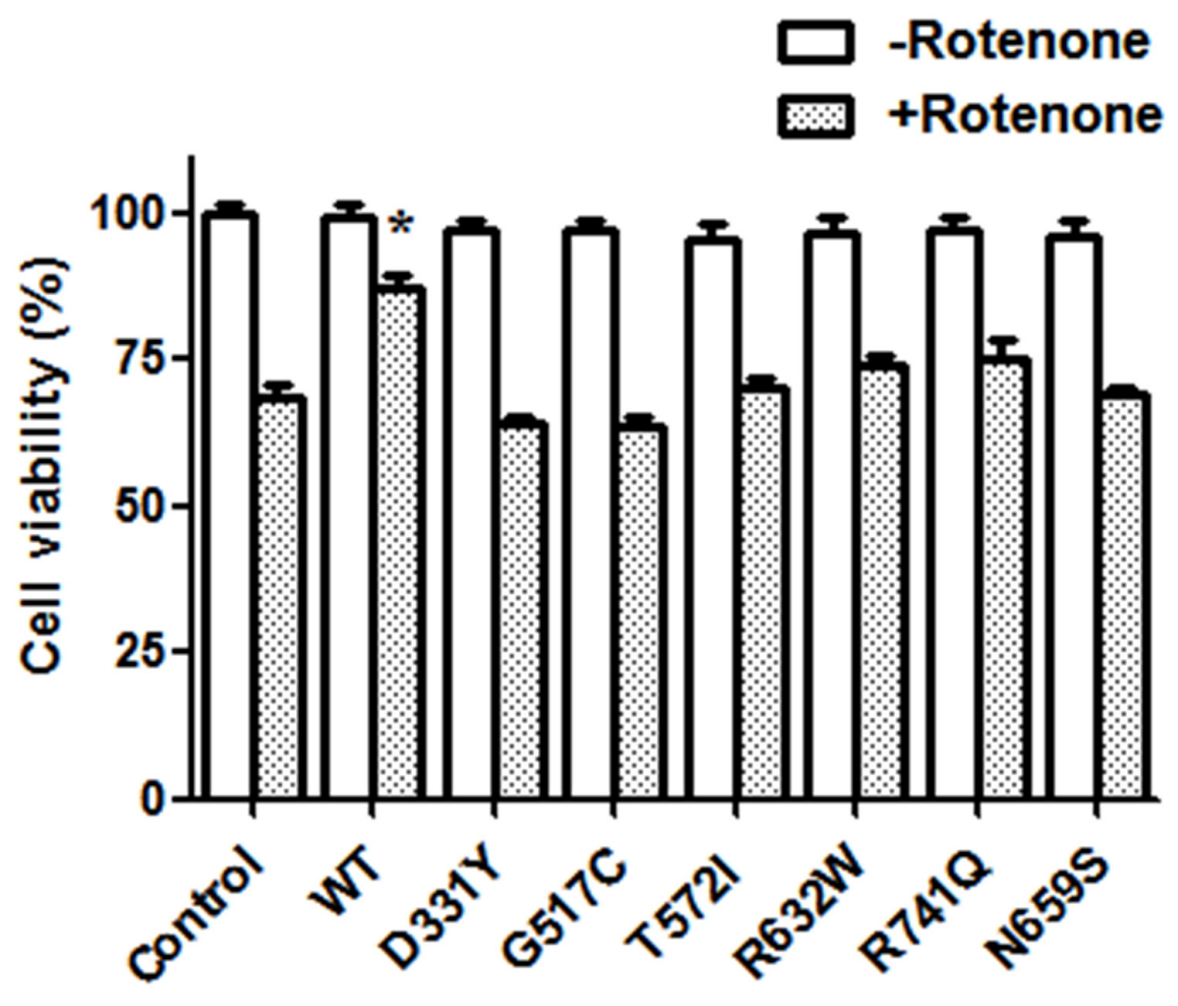
Expression of WT PLA2G6, but not PARK14 mutant PLA2G6, attenuates rotenone-induced death of SH-SY5Y dopaminergic cells Rotenone treatment (200 nM) for 24 hours caused a significant reduction of cell viability in control SH-SY5Y cells. Expression of WT PLA2G6 significantly prevented rotenone-induced cell death. In contrast, expression of PARK14 mutant PLA2G6 did not protect against rotenone-induced neurotoxicity. Each bar represents the mean ± SEM value of six independent experiments. *p< 0.05 compared to rotenone-treated control cells.

To study the effect of PARK14 mutations on the cytoprotective function of PLA2G6, SH-SY5Y dopaminergic cells expressing WT or PARK14 mutant PLA2G6 were treated 200 nM rotenone, an inhibitor of mitochondrial complex I, for 24 hours. Rotenone treatment decreased the cell viability of control SH-SY5Y cells (Figure 3). Expression of WT PLA2G6 exerted a neuroprotective effect by significantly reversing rotenone-induced cell death (Figure 3). Consistent with our hypothesis that PARK14 mutations cause the loss of function, PARK14 mutant (D331Y), (G517C), (T572I), (R632W), (N659S) or (R741Q) PLA2G6 failed to prevent rotenone-induced neurotoxicity (Figure 3).

### PARK14 PLA2G6 mutants are ineffective in preventing rotenone-induced activation of mitochondrial apoptotic pathway

**Figure 4:**
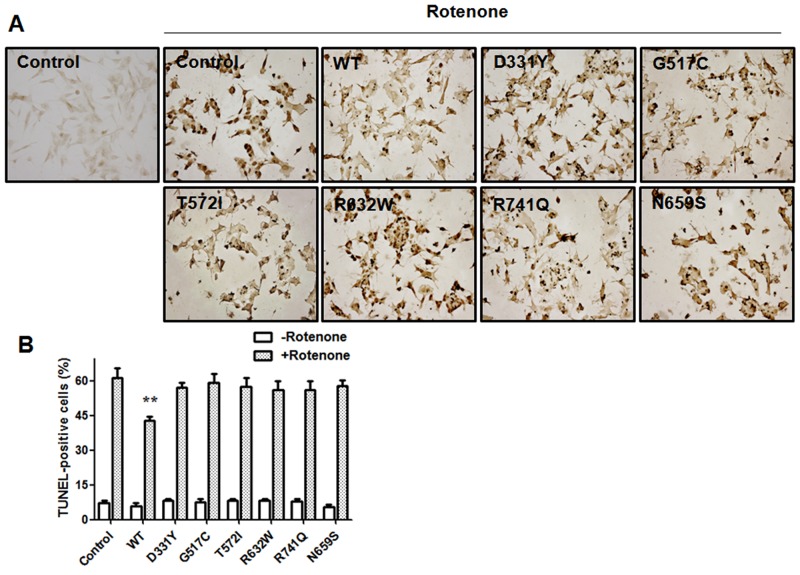
Expression of WT PLA2G6, but not PARK14 mutant PLA2G6, exerts a significant protective effect against rotenone-induced apoptotic death of SH-SY5Y dopaminergic cells **(A)** and **(B)** Treating SH-SY5Y cells with rotenone greatly increased the number of TUNEL-positive cells. In contrast to the expression of WT PLA2G6, PARK14 mutant PLA2G6 failed to prevent rotenone-induced apoptotic death of SH-SY5Y dopaminergic cells. Each bar represents the mean ± SEM value of six independent experiments. **p<0.01 compared to rotenone-treated control cells.

Activation of mitochondrial apoptotic cascade is believed to cause neuronal death observed in several neurodegenerative diseases including Parkinson’s disease [33]. Rotenone has been shown to induce the apoptotic neuronal death [28, 34, 35]. Terminal deoxynucleotidyl transferase (TdT)-mediated dUTP nick-end labeling (TUNEL) analysis indicated that rotenone treatment induced the apoptotic death of SH-SY5Y dopaminergic cells by greatly increasing the number of TUNEL-positive cells (Figure 4) [36–38]. Compared to rotenone-treated control SH-SY5Y cells, overexpression of WT PLA2G6 significantly decreased the number of TUNEL-positive SH-SY5Y cells. In contrast, expression of PARK14 mutant (D331Y), (G517C), (T572I), (R632W), (N659S) or (R741Q) PLA2G6 was ineffective in preventing rotenone-induced apoptotic death of SH-SY5Y dopaminergic cells (Figure 4).

**Figure 5:**
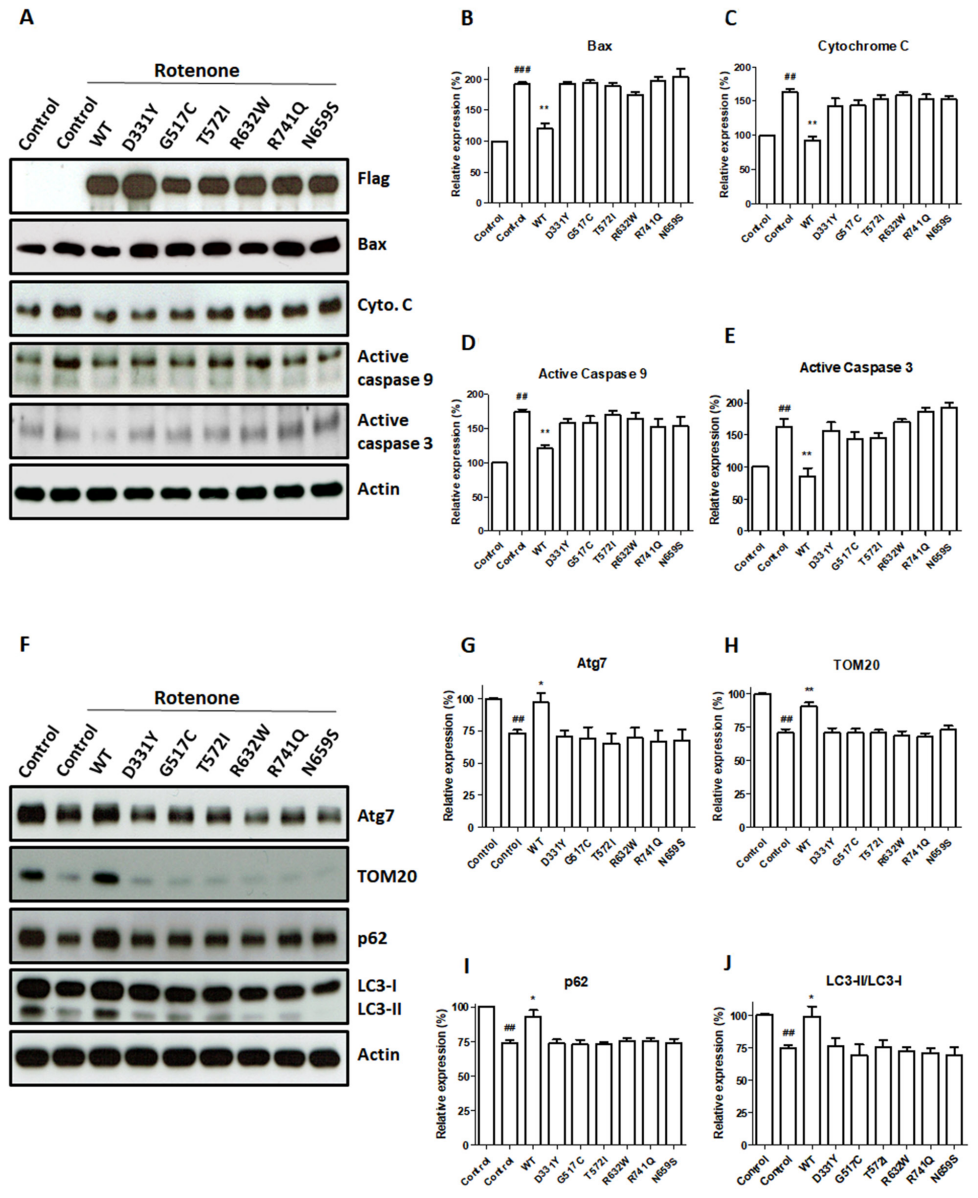
PARK14 PLA2G6 mutants fail to attenuate rotenone-induced upregulation of apoptotic protein levels and mitophagy impairment **(A)** Rotenone treatment significantly increased cytosolic protein levels of Bax, cytochrome c, active cleaved caspase-9 and active cleaved caspase-3 in control SH-SY5Y cells. Expression of WT PLA2G6 reversed rotenone-induced mitochondrial apoptotic cascade. In contrast, PARK14 mutant PLA2G6 failed to prevent rotenone-induced increase in cytosolic levels of Bax, cytochrome c, active caspase-9 and active caspase-3. **(B-E)** Level of apoptosis related proteins was quantified by densitometer. **(F)** Rotenone decreased the protein levels of Atg7, TOM20, p62 and LC3-II mitophagy proteins. Overexpression of WT PLA2G6 prevented rotenone-induced decrease in protein levels of Atg7, TOM20, p62 and LC3-II mitophagy markers. In contrast, expression of PARK14 mutant PLA2G6 failed to prevent rotenone-induced reduction of Atg7, TOM20, p62 and LC3-II proteins. **(G-J)** The expression level of mitophagy proteins was quantified by the densitometer. Each bar shows the mean ± SEM value of five independent experiments. ##p<0.01, ###p<0.001 compared to SH-SY5Y cells. *p<0.05, **p<0.01 compared to rotenone-treated control cells.

Rotenone exposure induced apoptotic cell death by increasing the cytosolic level of Bax and causing the subsequent release of cytochrome c from mitochondria, which led to the activation of caspase-9 and caspase-3 (Figure 5). In accordance with our finding that WT PLA2G6 prevented rotenone-induced apoptotic death of SH-SY5Y dopaminergic cells (Figure 4), expression of WT PLA2G6 significantly reversed rotenone-induced upregulation of protein level of cytosolic Bax, cytochrome c, active caspase-9 or active caspase-3 (Figure 5). In contrast to WT PLA2G6, PARK14 mutant (D331Y), (G517C), (T572I), (R632W), (N659S) or (R741Q) PLA2G6 was defective in preventing rotenone-induced activation of mitochondrial apoptotic cascade (Figure 5A–5E).

### PARK14 mutant PLA2G6 is ineffective in preventing rotenone-induced mitophagy impairment

Mitochondrial autophagy (also referred as mitophagy) selectively eliminates damaged mitochondria through autophagy pathway and protects cells from the damage of mitochondrial dysfunction and apoptosis induction [39]. Rotenone treatment reduces autophagic flux and impairs mitophagy prior to initiating cell death in SH-SY5Y dopaminergic cells [40]. To study the effect of WT or PARK14 mutant PLA2G6 on rotenone-induced mitophagy impairment, protein levels of mitophagy markers, including Atg7, TOM20, p62 and LC3-I/II, were examined in SH-SY5Y dopaminergic cells expressing WT or PARK14 mutant PLA2G6.

The level of Atg7, TOM20, p62 or LC3-II mitophagy protein was decreased in rotenone-treated SH-SY5Y dopaminergic neurons (Figure 5F–5J). The LC3-II/LC3-I protein ratio indicates the autophagic influx. Rotenone treatment reduced the protein ratio of LC3-II/LC3-I in SH-SY5Y cells (Figure 5F and 5J). Compared to rotenone-treated SH-SY5Y cells, expression of WT PLA2G6 increased the level of Atg7, p62, TOM20 or LC3-II (Figure 5F–5J). WT PLA2G6 ameliorated rotenone-induced decrease in the ratio of LC3-II/LC3-I (Figure 5F–5J). In contrast, expression of PARK14 mutant PLA2G6 was ineffective in reversing rotenone-induced reduction of Atg7, p62, TOM20 and LC3-II mitophagy markers (Figure 5F–5J). PARK14 PLA2G6 mutants failed to prevent rotenone-induced decrease in protein ratio of LC3-II/LC3-I (Figure 5F and 5J). Our results suggest that WT PLA2G6, but not PARK14 mutant PLA2G6, prevented rotenone-induced mitophagy impairment.

### PARK14 mutant PLA2G6 fails to reverse rotenone-induced loss of mitochondrial membrane potential (ΔΨm) and mitochondrial ROS generation

Our previous study showed that rotenone treatment causes the loss of mitochondrial membrane potential (ΔΨm) and mitochondrial ROS generation in SH-SY5Y dopaminergic cells [41]. PLA2G6 is believed to possess a neuroprotective effect by playing an important role in maintaining mitochondrial function [31, 32]. Thus, it is very likely that WT PLA2G6 prevents rotenone-induced loss of mitochondrial membrane potential (ΔΨm) and mitochondrial ROS generation.

**Figure 6:**
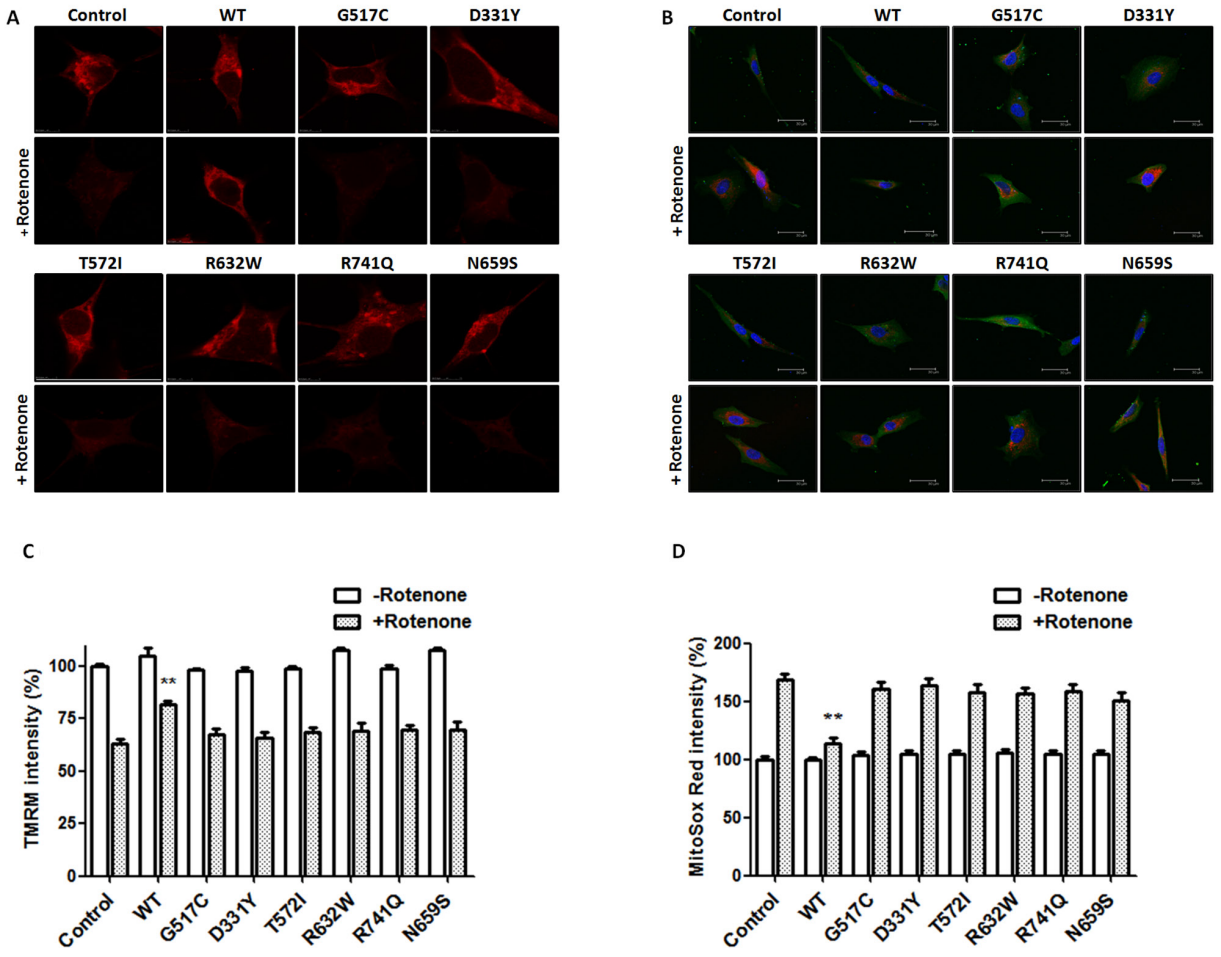
PARK14 mutant PLA2G6 is ineffective in preventing rotenone-induced loss of mitochondrial membrane potential and ROS production **(A, C)** SH-SY5Y dopaminergic cells were treated with 200 nM rotenone for 24 hours. Expression of WT PLA2G6 significantly prevented rotenone-induced reduction in TMRM fluorescence intensity and loss of ΔΨm. In contrast, PARK14 mutant PLA2G6 did not reverse rotenone-induced loss of TMRM fluorescence intensity and ΔΨm. **(B, D)** Expression of WT PLA2G6 significantly inhibited rotenone-induced increase in MitoSox Red fluorescence intensity and ROS generation. In contrast to WT PLA2G6, PARK14 mutant PLA2G6 failed to attenuate rotenone-induced ROS production. Scale bar is 30 μm. Each bar represents the mean ± SEM value of 25–35 cells. **p<0.01 compared to rotenone-treated control SH-SY5Y cells.

Confocal image of TMRM fluorescence was performed to visualize hyperpolarized ΔΨm of SH-SY5Y dopaminergic cells. Rotenone treatment caused a significant loss of ΔΨm in control SH-SY5Y cells (Figure 6A and 6C). Overexpression of WT PLA2G6 significantly attenuated rotenone-induced loss of TMRM fluorescence intensity and ΔΨm (Figure 6A and 6C). In contrast to WT PLA2G6, PARK14 mutant (D331Y), (G517C), (T572I), (R632W), (N659S) or (R741Q) PLA2G6 was ineffective in reversing rotenone-induced loss of ΔΨm (Figure 6A and 6C).

Confocal MitoSox Red imaging was performed to visualize mitochondrial level of superoxide anion, a major ROS produced by the mitochondria. MitoSox Red is selectively targeted to the mitochondria, and exhibits red fluorescence as a result of oxidation by superoxide anion. Rotenone treatment significantly increased MitoSox Red fluorescence intensity and ROS production in control SH-SY5Y cells (Figure 6B and 6D). Expression of WT PLA2G6 significantly prevented rotenone-induced increase in MitoSox Red fluorescence signal and ROS generation (Figure 6B and 6D). In contrast, PARK14 mutant (D331Y), (G517C), (T572I), (R632W), (N659S) or (R741Q) PLA2G6 was defective in inhibiting rotenone-induced ROS generation (Figure 6B and 6D).

### PARK14 PLA2G6 mutants fail to attenuate rotenone-induced reduction of mitochondrial complex I activity and intracellular ATP content

**Figure 7:**
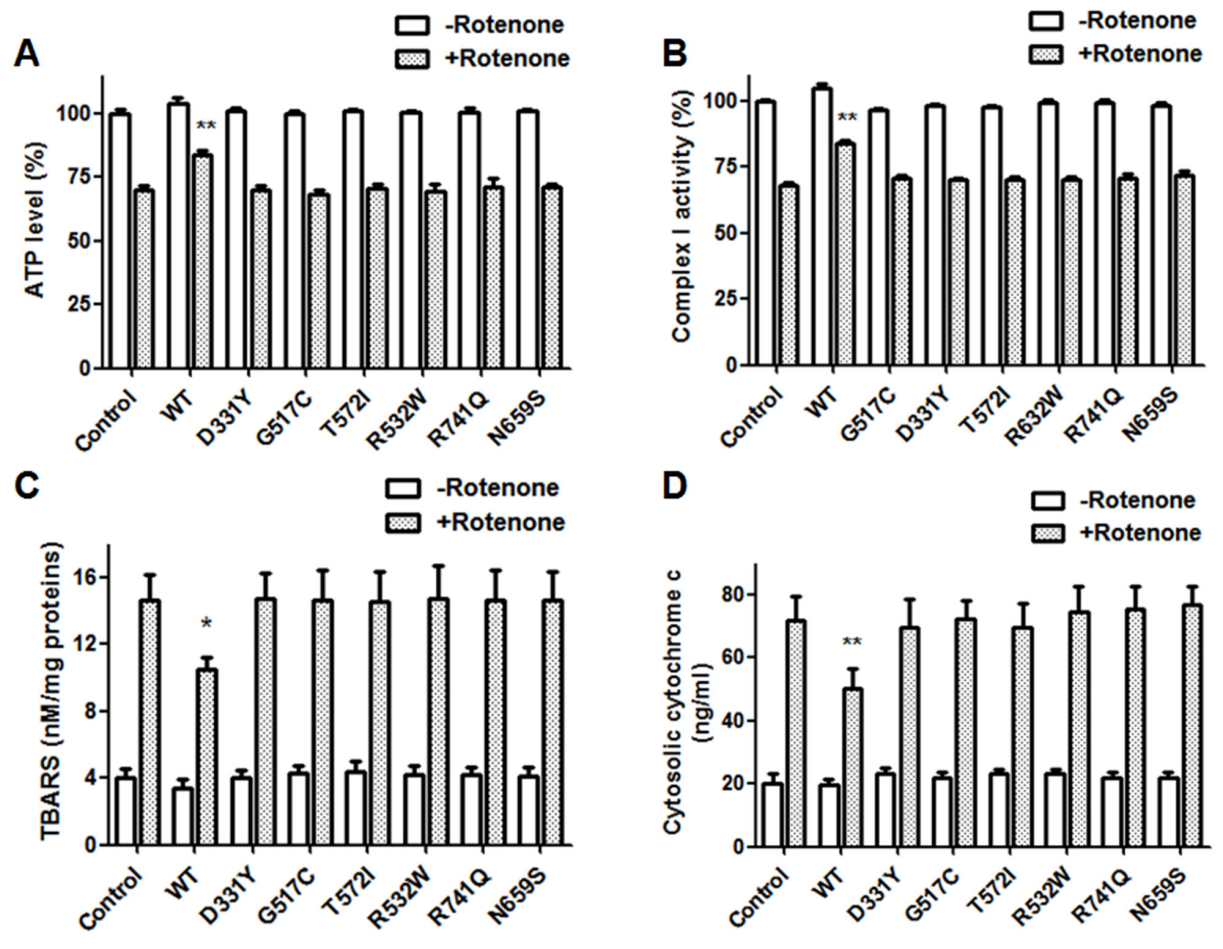
PARK14 mutant PLA2G6 fails to prevent rotenone-induced reduction of mitochondrial complex I activity, intracellular ATP content and rotenone-induced increase of mitochondrial lipid peroxidation and cytochrome c release Rotenone treatment decreased the level of intracellular ATP **(A)** and the activity of mitochondrial complex I **(B)**. The level of mitochondrial lipid peroxidation **(C)** and cytosolic cytochrome c release **(D)** was increased in rotenone-treated SH-SY5Y dopaminergic cells. Expression of WT PLA2G6 significantly inhibited rotenone-induced decrease in mitochondrial complex I activity and intracellular ATP content (A and B). In contrast, PARK14 PLA2G6 mutants were ineffective in preventing rotenone-induced reduction of mitochondrial complex I activity and intracellular ATP level (A and B). Overexpression of WT PLA2G6 significantly prevented rotenone-induced increase in mitochondrial lipid peroxidation and cytochrome c release (C and D). In contrast, PARK14 PLA2G6 mutants were ineffective in attenuating rotenone-induced increase of mitochondrial lipid peroxidation and cytochrome c release (C and D). Each bar shows the mean ± SEM value of five or six independent experiments. *p<0.05, **p<0.01 compared to rotenone-treated control cells.

Rotenone treatment decreased the activity of mitochondrial complex I and the level of intracellular ATP in SH-SY5Y dopaminergic cells (Figure 7A and 7B). Consistent with previous studies showing that PLA2G6 plays an important role in maintaining mitochondrial function [31, 32], overexpression of WT PLA2G6 prevented rotenone-induced decrease in mitochondrial complex I activity and intracellular ATP level (Figure 7). In contrast to WT PLA2G6, expression of PARK14 mutant (D331Y), (G517C), (T572I), (R632W), (N659S) or (R741Q) PLA2G6 was defective in reversing rotenone-induced reduction of mitochondrial complex I activity and intracellular ATP content (Figure 7A and 7B).

### PARK14 mutant PLA2G6 is ineffective in preventing rotenone-induced increase of mitochondrial lipid peroxidation and cytochrome c release

Cardiolipin, a phospholipid of inner mitochondrial membrane, mediates cytochrome c release and is important in maintaining mitochondrial function. Reactive oxygen species (ROS) generated from mitochondria leads to cardiolipin oxidation and subsequent activation of apoptosis through the release of cytochrome c [42]. Simultaneous measurements of mitochondrial lipid peroxidation and cytochrome c release can be used to evaluate the level of cardiolipin oxidation. Increased level of mitochondrial lipid peroxidation and cytosolic cytochrome c reflects mitochondrial cardiolipin oxidation [42, 43]. The thiobarbituric acid reactive substances (TBARS) assay was used to assess mitochondrial lipid peroxidation. Mitochondrial lipid peroxidation and cytosolic cytochrome c release were increased in rotenone-treated SH-SY5Y dopaminergic neurons (Figure 7C and 7D). Expression of WT PLA2G6 ameliorated rotenone-induced increase in mitochondrial lipid peroxidation and cytochrome c release (Figure 7C and 7D). In contrast, PARK14 mutant (D331Y), (G517C), (T572I), (R632W), (N659S) or (R741Q) PLA2G6 was defective in attenuating rotenone-induced increase of mitochondrial membrane peroxidation and cytosolic cytochrome c release (Figure 7C and 7D). Our results suggest that WT PLA2G6, but not PARK14 mutant PLA2G6, prevents rotenone-induced cardiolipin oxidation through reducing mitochondrial lipid peroxidation and cytochrome c release.

## DISCUSSION

Ca^2+^-independent phospholipase A_2_ group 6 (PLA2G6) is ubiquitously expressed, particularly in all regions of the mammalian brain [44]. PLA2G6 belongs to a family of phospholipase A_2_ that hydrolyze phospholipid at the sn-2 position and generate free fatty acids and lysophospholipids. PLA2G6 plays an important role in cell membrane homeostasis [45]. The release of fatty acid, usually arachidonic acid, can be converted into eicosanoids, including prostaglandins, leukotrienes and lipoxins, which initiate intracellular signaling pathway [46]. PLA2G6 is also involved in regulating various cellular functions including remodeling of membrane phospholipids, calcium signaling, cell growth, mitochondrial function and apoptosis [31, 45–49].

Mutations of PLA2G6 gene cause NBIA2 and PARK14 [50]. Mutations of PLA2G6 identified in patients with NBIA2 are frequently large deletions [51, 52]. PARK14 mutations of PLA2G6 are homozygous and compound-heterozygous missense mutations [10–12, 15–19].

PARK14 patients display autosomal recessive inheritance and early-onset dystonia-parkinsonism [7–10]. Several missense mutations of PLA2G6, including (D331Y), (G517C), (T572I), (R632W), (N659S) and (R741Q) PLA2G6, were observed in PARK14 patients [7, 15–17, 19–21]. (G517C) and (R632W) PLA2G6 were also found in patients with NBIA2 [11, 15, 21, 53]. In the present study, we investigated the possible pathogenic mechanism of PARK14 mutant PLA2G6-induced PD using rotenone-induced cellular model of PD.

In accordance with a previous study [31], our results showed that wild-type (WT) PLA2G6 is expressed in the mitochondria. Knockout of PLA2G6 gene in the *Drosophila* or mouse led to mitochondrial dysfunction, oxidative stress and abnormality of mitochondrial membrane [32, 48]. Mitochondrial dysfunction and subsequent oxidative stress result in the generation of ROS. Overproduction of ROS promotes the peroxidation of mitochondrial phospholipids. Oxidized phospholipid promotes the formation of reactive aldehydes, which are neurotoxic intermediate products and cause neuronal death. Mitochondrial phospholipid cardiolipin is involved in maintaining the structure of mitochondrial inner membrane and stabilization of respiratory chain supercomplexes [54]. Accumulation of oxidized cardiolipin results in the release of cytochrome c and leads to the activation of apoptotic death pathway [42]. PLA2G6 participates in the remodeling of cardiolipin and repairs oxidized cardiolipin [43]. Therefore, mitochondrial PLA2G6 exerts a neuroprotective effect against various cellular stresses by maintaining mitochondrial function. The level of cardiolipin oxidation can be evaluated by simultaneously measuring mitochondrial lipid peroxidation and cytosolic cytochrome c release [42, 43]. In the present study, rotenone-induced increased level of mitochondrial lipid peroxidation and cytochrome c reflects the oxidation of mitochondrial cardiolipin [42, 43]. In accordance with the hypothesis that PLA2G6 prevents cardiolipin oxidation and protects against the activation of apoptotic death pathway, WT PLA2G6, but not PARK14 mutant PLA2G6, attenuates rotenone-induced cardiolipin oxidation through inhibiting mitochondrial lipid peroxidation and cytochrome c release.

Mitophagy selectively eliminates damaged mitochondria through autophagy pathway and protects cells from the damage of mitochondrial dysfunction and apoptosis induction [39]. PLA2G6 is crucial in maintaining mitochondrial health [31]. Deficiency of PLA2G6 leads to mitochondrial dysfunction [32]. In the present study, expression of WT PLA2G6 prevents rotenone-induced decrease in protein level of Atg7, p62, TOM20 and LC3-II mitophagy markers, suggesting that WT PLA2G6 attenuates rotenone-induced mitophagy impairment. In contrast, PARK14 mutant PLA2G6 fails to prevent rotenone-induced mitophagy impairment.

Activation of mitochondrial apoptotic pathway causes neuronal death observed in several neurodegenerative diseases including Parkinson’s disease [33]. In the present study, rotenone, an inhibitor of mitochondrial complex I, induced the apoptotic death of SH-SY5Y dopaminergic cells by causing the activation of mitochondrial apoptotic cascade, such as an increase in cytosolic protein levels of Bax, cytochrome c, active caspase 9 and active caspase 3 [27, 30, 55]. We hypothesized that WT PLA2G6 exerts anti-apoptotic and neuroprotective effects on dopaminergic neurons. Consistent with our hypothesis, expression of WT PLA2G6 prevented rotenone-induced apoptotic death of SH-SY5Y dopaminergic cells and protein upregulation of Bax, cytochrome c, active caspase-9 or active caspase-3 in the cytosol.

Treating SH-SY5Y dopaminergic cells with rotenone has been shown to cause the loss of mitochondrial membrane potential (ΔΨm) and mitochondrial ROS generation [41], which could lead to the activation of mitochondrial apoptotic pathway. PLA2G6 is believed to play an important role in maintaining mitochondrial function [31, 32]. As a result, expression of WT PLA2G6 significantly prevented rotenone-induced loss of ΔΨm and ROS production in SH-SY5Y dopaminergic cells. Overexpression of WT PLA2G6 in SH-SY5Y cells also reversed rotenone-induced decrease in mitochondrial complex I activity and intracellular ATP content.

Autosomal recessive inheritance suggests the involvement of loss of PLA2G6 function in PARK14 pathogenesis. In accordance with this hypothesis, phospholipase A_2_ activity assays showed that compared to WT PLA2G6, PARK14 mutant (D331Y), (G517C), (T572I), (R632W), (N659S) or (R741Q) PLA2G6 possessed a significantly reduced activity of phospholipase A_2_. Therefore, we hypothesized that PARK14 mutations cause the loss of PLA2G6 function and impair the ability of PLA2G6 to exert an anti-apoptotic effect and maintain mitochondria function. Consistent with our hypothesis, PARK14 mutant (D331Y), (G517C), (T572I), (R632W), (N659S) or (R741Q) PLA2G6 was defective in preventing rotenone-induced apoptotic death of SH-SY5Y dopaminergic cells and activation of mitochondrial apoptotic pathway. PARK14 PLA2G6 mutants also failed to inhibit rotenone-induced loss of ΔΨm and mitochondrial ROS generation. Similar to WT PLA2G6, PARK14 mutant (D331Y), (G517C), (T572I), (R632W), (N659S) or (R741Q) PLA2G6 is also expressed in the mitochondrial fraction, suggesting that impaired cytoprotective effect of PARK14 PLA2G6 mutants is not caused by a failed expression of mutant PLA2G6 in the mitochondria.

Mutations of PTEN-induced kinase 1(PINK1) gene, which encodes a mitochondrial Ser/Thr protein kinase, cause the autosomal recessive familial type 6 of Parkinson’s disease (PARK6) [9, 10]. Mitochondrial PINK1 exerts anti-apoptotic and neuroprotective effects by playing an important role in maintaining mitochondrial function and integrity [56]. Our previous study showed that WT PINK1 blocks mitochondrial release of apoptogenic cytochrome c and exerts anti-apoptotic effect by inhibiting the opening of mitochondrial permeability transition pore (mPTP) and that PARK6 mutant PINK1 loses its ability to prevent mPTP opening and cytochrome c release [57]. WT PINK1 is required for maintaining a hyperpolarized mitochondrial membrane potential (ΔΨm) and mitochondrial morphology of dopaminergic neurons. In contrast, PARK6 mutant PINK1 fails to maintain a hyperpolarized ΔΨm and mitochondrial morphology [58]. The results of present study suggest that similar to PARK6 mutation-induced loss of PINK1 function, PARK14 mutations cause the loss of PLA2G6 function and impair the ability of PLA2G6 to maintain mitochondrial function and exert an anti-apoptotic effect on dopaminergic cells.

PLA2G6 is not only involved in the pathogenesis of PARK14-linked parkinsonism but also in idiopathic PD [59, 60]. Mutation of PLA2G6 was found in patients affected with PARK14 or idiopathic PD [17]. The neuropathology of PARK14 is similar to that of idiopathic PD [60]. The accumulation of PLA2G6 is detected in Lewy bodies observed from patients with PARK14 and idiopathic PD [59]. Since genetic PD and idiopathic PD patients exhibit similar clinical and neuropathological features, it is likely that common molecular mechanisms are involved in the pathogenesis of PARK14 and idiopathic PD [9, 61]. Consistent with this hypothesis, mitochondrial dysfunction and oxidative stress play an important role in the pathogenesis of PARK14 and idiopathic PD [22, 23].

In summary, the present study suggests that WT PLA2G6 exerts a neuroprotective effect by maintaining mitochondrial function and preventing activation of mitochondrial apoptotic pathway. In contrast, PARK14 mutant (D331Y), (G517C), (T572I), (R632W), (N659S) or (R741Q) PLA2G6 loses its ability to maintain mitochondrial function and is defective inpreventing mitochondrial dysfunction, ROS generation and activation of mitochondrial apoptotic pathway. Future study using a knockin mouse model of PARK14 is required to confirm the molecular pathogenic mechanism of PARK14 mutant PLA2G6 observed in the cellular model of PD.

## MATERIALS AND METHODS

### Preparation of cDNA encoding mutant PLA2G6

Oligonucleotide-directed mutagenesis using PCR amplification was performed to prepare cDNA encoding PARK14 mutant PLA2G6 as previously described [62]. PLA2G6 mutations were verified by Sanger sequence analysis. The cDNA of wild-type (WT), (D331Y), (G517C), (N569S) (T572I), (R632W) or (R741Q) PLA2G6 was subcloned into a mammalian expression vector pcDNA3 (Invitrogen) containing the FLAG-tag sequence (DYKDDDDK).

### Cell culture, transfection and rotenone treatment

SH-SY5Y cells express tyrosine hydroxylase and synthesize dopamine [63]. Human SH-SY5Y dopaminergic cells were grown in Dulbecco’s modified Eagle’s medium supplemented with nutrient mixture F-12 (Ham) (1:1, v/v) (Gibco), 2.4 g/L sodium bicarbonate (Sigma-Aldrich), 100 units/ml penicillin, 100 μg/ml streptomycin and 10 % fetal bovine serum. Cells were grown at 37°C in a humidified air with 5% CO_2_. SH-SY5Y dopaminergic cells were transfected with the cDNA of WT or PARK14 mutant PLA2G6 using X-tremeGENE HP DNA Transfection Reagent (Cat. 6366244001, Roche) according to the manufacturer’s instructions. 24 hours after transfection, SH-SY5Y cells were treated with 200 nM rotenone.

### Subcellular fractionation

To analyze the subcellular expression of PLA2G6, cytosolic and mitochondrial fractions of SH-SY5Y cells expressing WT or mutant PLA2G6 were prepared. Briefly, cells were lysed in an ice-cold sucrose buffer (210 mM mannitol, 70 mM sucrose, 10 mM HEPES, pH7.3, 1 mM EGTA, 1 mM DTT, 5 μg/ml pepstatin, 5 μg/ml leupeptin, 5 μg/ml aprotinin, and 0.3 mM PMSF) and homogenized using a Dounce homogenizer on ice. Mitochondrial fraction was collected by centrifugation at 9500×g for 9 min at 4 °C. The resulting supernatant was further centrifuged at 16,000×g for 20 min at 4 °C, and the final supernatant was used as cytosolic fraction. Protein concentrations were determined using a modified Lowry assay (DC Protein Assay Kit Cat. 5000112, Bio-Rad). Protein samples (30 μg) of mitochondrial or cytosolic extracts were separated on a 12% SDS polyacrylamide gel and transferred to PVDF membrane. The membrane was incubated at 4°C overnight with anti-FLAG monoclonal antiserum (Cat.F3165, Sigma-Aldrich). Then, FLAG-tagged PLA2G6 on the membrane was visualized by using the ECL protocol (Cat.WBKLS0500, Millipore). To verify the purity of mitochondrial fraction and detect possible mitochondrial contamination of cytosolic extract, membrane was stripped and reblotted with monoclonal anti-cytochrome c oxidase subunit IV (COX-IV) antibody, a mitochondrial marker (Cat. MA5-15078, Thermo Fisher Scientific). We verified that COX-IV was present in the mitochondrial extract and not detected in the cytosolic fraction.

### Immunofluorescence staining

Two days after the transfection, transfected SH-SY5Y cells were fixed with 4% paraformaldehyde for 10 minutes and then permeabilized with 0.5% Triton X-100 in PBS. After washing three times, fixed cells were blocked with 5% FBS and incubated with anti-FLAG and anti-COX IV primary antibodies. The cells were then stained with Alexa Fluor 488- and Alexa Fluor 594-conjugated secondary antibodies (Cat.A-11001 and Cat.A-21207, Thermo Fisher Scientific) for 1 hour at room temperature. The fluorescence was visualized using a Leica DM6000 microscope equipped with Leica TCS SP5 confocal spectral scanning system.

### Measurement of calcium-independent phospholipase A_2_ (iPLA_2_) activity

The phospholipase A_2_ activity of PLA2G6 was determined by using a modified commercial kit originally used for cPLA_2_ (cPLA_2_ assay kit, Cat. 765021, Cayman Chemicals) and measured as previously described [64, 65]. Briefly, proteins was extracted with a Ca^2+^-free CHAPS buffer (0.1% CHAPS, 50 mM HEPES, pH 7.4, 4 mM EDTA, 5 μg/ml pepstatin, 5 μg/ml leupeptin, 5 μg/ml aprotinin, and 0.3 mM PMSF), followed by centrifugation at 14000×g for 20 minutes at 4°C. To examine iPLA_2_ activity instead of cPLA_2_, protein extracts were incubated with a synthetic substrate, arachidonoyl thio-phosphatidylcholine for 1 h at 25 °C in a modified Ca^2+^ free buffer (4 mM EGTA, 160 mM HEPES, pH 7.4, 300 mM NaCl, 8 mM Triton X-100, 60% glycerol, and 2 mg/mL bovine serum albumin). iPLA_2_ hydrolyzed arachidonoyl thio-phosphatidylcholine and then released free thiols. The production of free thiols was measured by adding 5,5 o-dithiobis, 2-nitrobenzoic acid, and iPLA_2_ activity was determined by measuring the absorbance at 405 nm. The activity of iPLA_2_ was expressed as nmol/min/mg of protein.

### Assay of cell survival

Cell Counting Kit-8 (Cat.96992, Fluka-Sigma-Aldrich) was used to assess the cell viability of SH-SY5Y dopaminergic cells transfected with the cDNA of WT or PARK14 mutant PLA2G6. Briefly, a total of 1×10^4^ transfected SH-SY5Y cells were seeded per well in a 96-well plate, and cells were administered with 200 nM rotenone. After 24-hour incubation, WST-8 [2-(2-methoxy-4-nitrophenyl)-3-(4-nitrophenyl)-5-(2,4-disulfophenyl)-2H-tetrazolium, monosodium salt] was added into each well in a humidified 5% CO_2_ atmosphere at 37°C for 1 hour. The optical density (OD) was measured with an xMark microplate absorbance spectrophotometer (Bio-Rad) at 450 nm.

### Terminal deoxynucleotidyl transferase (TdT)-mediated dUTP nick-end labeling (TUNEL) staining

TUNEL staining (In Situ Cell Death Detection Kit, Cat. 11684817910, Roche) was performed according to the manufacture’s recommendation [36–38]. Briefly, 5×10^5^ SH-SY5Y cells transfected with the cDNA of WT or mutant PLA2G6 were seeded on coverslips in 6-well plates and treated with 200 nM rotenone for 24 hours. Then, SH-SY5Y cells were fixed in 4% paraformaldehyde for 10 min, washed with PBS and permeabilized with 0.1% sodium citrate and 0.1% Triton X-100 for 10 min at room temperature. Subsequently, SH-SY5Y cells were incubated with TUNEL reaction mixture for 1h at 37°C, followed by incubation with Converter-POD for 30 min at 37°C. After washing, cells were visualized using 3’,3’-diaminobenzidine (DAB) detection system (Cat.SK-4100, Vector Laboratories) for 10 min at room temperature. The images were obtained using an Eclipse 80i microscope (Nikon).

### Western blot

Cytosolic or mitochondrial fraction of SH-SY5Y dopaminergic cells was prepared as described above. Subsequently, 30 μg of cytosolic or mitochondrial protein was separated on 12% SDS-polyacrylamide gel and transferred to PVDF membrane. Then, the membrane was incubated at 4 °C overnight with one of the following diluted primary antibodies: (1) Polyclonal anti-cleaved active caspase 9 antibody (Cat.9508, Cell Signaling Technology). (2) Polyclonal anti-cleaved active caspase 3 antiserum (Cat.9662, Cell Signaling Technology). (3) Polyclonal anti-Bax antibody (Cat.2772, Cell Signaling Technology). (4) Polyclonal anti-cytochrome c antiserum (Cat. ab13575, Abcam). (5) Monoclonal anti-TOM20 antibody (Cat.42406, Cell Signaling Technology). (6) Polyclonal anti-Atg7 antiserum (Cat.2631, Cell Signaling Technology). (7) Monoclonal anti-p62 antibody (Cat.8025, Cell Signaling Technology). (8) Polyclonal anti-LC3-I/II antiserum (Cat.4108, Cell Signaling Technology). After the wash, the membrane was incubated with anti-rabbit secondary antibody conjugated with horseradish peroxidase (HRP). Then, immunoreactive proteins were visualized by using an ECL kit. Relative protein expressions were normalized with the level of β-actin using a densitometer (Molecular Dynamics Model 375A).

### Determination of mitochondrial membrane potential (ΔΨm)

For the determination of mitochondrial membrane potential (ΔΨm), SH-SY5Y dopaminergic cells were loaded with a potential sensitive dye TMRM (tetramethylrhodamine methyl ester; 100 nM; T668, Molecular Probes, Thermo Fisher Scientific) in HEPES buffered saline (NaCl 140 mM, KCl 5 mM, MgCl_2_ 1 mM, CaCl_2_ 2 mM, glucose 10 mM, HEPES 5 mM, pH 7.3) for 10 min at 37 °C. SH-SY5Y cells were then washed with HEPES buffered saline, and transferred to the recording chamber mounted on a Leica DM6000 microscope equipped with Leica TCS SP5 confocal spectral scanning system. TMRM was excited at 543 nm with a HeNe green laser, and the emitted fluorescent signal at 560-620 nm was collected. Z-stacks of 15 confocal TMRM fluorescence images were processed and analyzed by LAS AF software (Leica) as previously described [41].

### Measurement of mitochondrial superoxide

MitoSOX Red selectively targeted to the mitochondria is oxidized by superoxide and exhibits red fluorescence. Confocal MitoSOX Red staining was performed as described previously [41]. Briefly, SH-SY5Y dopaminergic cells were incubated with 5 μM MitoSOX Red (Cat.M36008, Molecular Probes, Thermo Fisher Scientific) for 10 min at 37 °C. After washout, MitoSOX Red was excited at 514 nm with an Ar-blue laser, and fluorescence signal was detected at 540-620 nm emission. MitoSOX Red fluorescence images were analyzed by Leica LAS AF software.

### Analysis of mitochondrial complex I activity

Mitochondrial complex I enzyme activity was measured using complex I enzyme activity assay kit according to the manufacturer’s instructions (Cat.ab109721, Abcam). Briefly, 50 μg of mitochondrial protein was loaded into the well of microplate coated with complex I capture antibody, and incubated for 3 hours at room temperature. The activity of complex I was measured following the oxidation of NADH to NAD^+^, which leads to increased absorbance at 450 nm. Complex I activity are expressed as the change in absorbance per minute per microgram protein.

### Measurement of intracellular ATP content

The content of cellular ATP levels was measured by using Luminescent ATP determination kit (Cat.A22066, Thermo Fisher Scientific) according to the manufacture’s protocol. Briefly, 10 μl of cell extracts or ATP standard reaction solutions, ranging from 100 nM to 5 μM, were added into 96-well luminescence assay plates. Then, 90 μl of reaction buffer was added into each well. Luminescence was measured with a fluorescence microplate Reader (TECAN Infinite M200 Pro) at 560-nm absorbance. Cellular ATP content was calculated according to ATP standard curve.

### Determination of cytochrome *c* release

The release of cytochrome c was examined using Cytochrome c ELISA Assay Kit according to manufacturer’s instructions (Cat.KHO1051, Thermo Fisher Scientific). Briefly, 100 μl of cytosolic or mitochondrial protein extracts was loaded into the well of microplate coated with a monoclonal antibody specific for human cytochrome c, followed by adding biotin conjugate for 2 hours at room temperature. After washing, streptavidin-HRP working solution was added to the wells and incubated for 1 hour at room temperature. Tetramethylbenzidine (TMB) substrate solutions were added to the wells and incubated for 15 minutes at room temperature in the dark. After addition of the stop solution, the absorbance was measured using an xMark microplate absorbance spectrophotometer (Bio-Rad) at 450 nm. All samples and the standards were analyzed in duplicate and the average of the duplicates was used for analysis.

### Measurement of mitochondrial membrane peroxidation

Lipid peroxidation of mitochondria was determined by evaluating the level of thiobarbituric acid reactive substances (TBARS) using the TBARS assay kit according to the manufacture’s protocol (Cat.10009055, Cayman Chemicals). Malondialdehyde (MDA), a marker of lipid peroxidation, interacts with thiobarbituric acid (TBA) and forms the MDA-TBA adduct under high temperature and acidic conditions. Briefly, 20 μl of mitochondrial protein extracts or standards was mixed with TBA color reagent and incubated at 100 °C for 1 hour. The level of MDA-TBA adduct was determined by measuring the absorbance at 540 nm with an xMark microplate absorbance spectrophotometer (Bio-Rad).

### Statistical analysis

All results were expressed as the mean ± SEM value of n experiments. Statistical significance among multiple experimental groups was determined by one-way ANOVA followed by post-hoc Tukey’s multiple comparison test. Unpaired student’s *t*-test (two-tailed) was used to determine the significant difference between two groups of data. A *p* value < 0.05 was considered significant.
